# Fluticasone propionate in chronic rhinosinusitis with nasal polyps (CRSwNP): an artificial intelligence-driven consensus

**DOI:** 10.3389/falgy.2025.1594655

**Published:** 2025-05-29

**Authors:** Emilio Avallone, Raffaella Iannella, Antonella Di Lullo, Michele Grasso, Salvatore Musto, Enzo Piermichele Troncone, Giuseppe Tortoriello, Bernardino Cassiano, Simona Nappi, Gianluca Bava, Giovanna Piazzetta, Giovanni Tomacelli, Aurelio D’Ecclesia, Giacomo Spinato, Giacomo Matrone, Doriano Politi, Carlo De Luca, Claudio Caporale, Livio Presutti, Gabriele Molteni, Ernesto Pasquini, Francesco Panu, Simonetta Masieri, Stefano Di Girolamo, Giulio Cesare Passali, Luca de Campora, Giandomenico Maggiore, Domenico Di Maria

**Affiliations:** ^1^ Department of Otolaryngology, AORN “San Pio”, Benevento, Italy; ^2^ CEO, Butterfly Decisions S.r.l., Salerno, Italy; ^3^ Department of Otolaryngology, AORN “Ospedale dei Colli”, Napoli, Italy; ^4^ Department of Otolaryngology, Asl NA 3- Nola (NA), Nola, Italy; ^5^ Simple Departmental Operational Unit of ENT, G. Paolo II Hospital - Lamezia Terme (CZ), Lamezia Terme, Italy; ^6^ Department of Medical and Surgical Sciences, Department of Otolaryngology, “Renato Dulbecco” University Hospital, Catanzaro, Italy; ^7^ Department of General and Specialized Surgery, Department of Otolaryngology, ASL Lecce Vito Fazzi Hospital, Lecce, Italy; ^8^ Department of Otolaryngology, Irccs Casa Sollievo Della Sofferenza San Giovanni Rotondo, Foggia, Italy; ^9^ Department of Neurosciences, Otolaryngology Unit, University of Padova, Padova, Italy; ^10^ Head of Data Science and AI, Butterfly Decisions, Salerno, Italy; ^11^ Department of Otolaryngology, “Ospedale Dell’Angelo Mestre ULSS3 Serenissima”, Mestre, Italy; ^12^ Fisioter Medical Center, ENT Division, Montecilfone Campobasso, Italy; ^13^ Department of Otolaryngology, “Santo Spirito Hospital”, Pescara, Italy; ^14^ Department of Otolaryngology-Head and Neck Surgery, IRCCS Azienda Ospedaliero-Universitaria Di Bologna, Bologna, Italy; ^15^ Alma Mater Studiorum-Università di Bologna, Bologna, Italy; ^16^ Department of Specialist Surgery, ENT Unit, “Bellaria” Hospital, Bologna, Italy; ^17^ Department of Otolaryngology, Policlinico Città di Quartu, Korian Kinetica Sardegna, Quartu, Italy; ^18^ Scienze Odontostomatologiche e Maxillo Facciale Department, Sapienza University, Roma, Italy; ^19^ Otolaryngology Department, Tor Vergata University, Rome, Italy; ^20^ Unit of Otorhinolaryngology and Head-Neck Surgery, “A. Gemelli” Hospital Foundation IRCCS, Rome, Italy; ^21^ Department of Otolaryngology, Azienda Ospedaliera S. Giovanni Addolorata, Roma, Italy; ^22^ Department Otolaryngology Head and Neck Surgery, AOU Careggi Firenze, Firenze, Italy

**Keywords:** CRSwNP, fluticasone, butterfly, consensus, nasal drops

## Abstract

**Introduction:**

Fluticasone propionate (FP) is a topical corticosteroid used to treat rhinosinusitis with nasal polyposis (CRSwNP). However, the need for a consensus on its use stems from the increasing focus on optimizing topical therapies to improve clinical outcomes and minimize systemic side effects.

**Materials and methods:**

The Butterfly Decisions AI platform facilitated the collection and integration of evaluations and feedback, facilitating an expert consensus on 13 statements.

**Results:**

The participants agreed highly on the different statements. The experts agreed that FP effectively reduces the need for surgery and controls the symptoms of CRSwNP. The use of advanced delivery systems significantly improved drug delivery and therapeutic outcomes. Treatment with FP was associated with a reduction in the recurrence of nasal polyps and an improvement in the patient's quality of life.

**Conclusions:**

FP, as other equal corticosteroids, represents a first-line local therapy for patients with CRSwNP without complicating comorbidities due to its high efficacy and low systemic bioavailability. The Butterfly Decisions platform has demonstrated the effectiveness of integrating AI tools into clinical decision-making, improving the transparency and objectivity of assessments.

## Introduction

Chronic rhinosinusitis, with or without nasal polyps (CRSwNP and CRSsNP), is an inflammatory syndrome affecting the nasal cavities and paranasal sinuses. It is characterized by persistent symptoms such as nasal congestion or obstruction, rhinorrhoea or retronasal discharge, facial pain or pressure and a reduced or lost sense of smell (hyposmia or anosmia). In patients with CRSwNP, the presence of bilateral polyps is typically localized in the ostiomeatal complex, contributing to obstruction and worsening of the clinical picture. Current guidelines for managing nasal polyposis recommend a multimodal approach, including pharmacological therapies and surgical intervention in severe cases. Intranasal corticosteroids represent the mainstay of drug therapy, reducing inflammation and the size of polyps, thus improving symptoms and respiratory function. Topical corticosteroids administered nasally are currently the first-line pharmacological treatment for the initial management of chronic rhinosinusitis with nasal polyps (CRSwNP). In addition, they are also used in post-operative management, helping to reduce residual inflammation, prevent recurrences, and maintain the benefits obtained with surgery (1–3).

In refractory patients, the use of oral corticosteroids or the use of targeted biological therapies, such as monoclonal antibodies (1, 2, 4), Functional endoscopic surgery of the paranasal sinuses (ESS) is indicated when medical therapy fails to control symptoms or in cases of complications (1). It is crucial to emphasize that surgery is not a definitive cure and requires ongoing post-operative management with intranasal corticosteroid drugs.

Among intranasal corticosteroids, fluticasone propionate (FP) stands out for its efficacy and safety profile. It binds to glucocorticoid receptors in nasal mucosa cells, inhibiting the release of inflammatory mediators such as cytokines and leukotrienes, significantly reducing inflammation and adequate symptom control (5). Demirel et al. showed that FP nasal drops compared with nasal spray produced the greatest effect in reducing the size of nasal polyps and improving symptoms (6).

A consensus statement was organized to optimize the use of FP in nasal polyposis and define standardized recommendations. However, an innovative element characterized this: the use of a platform powered by artificial intelligence to support decision-making. Butterfly Decisions, designed for AI-assisted decision-making, integrates advanced algorithms with expert knowledge to facilitate consensus-building processes while ensuring transparency and scientific rigor. The introduction of artificial intelligence was motivated by the need to overcome the limitations of traditional methods, particularly the authority bias and the influence of high-profile experts who could bias the consensus. Butterfly Decisions addresses these challenges by structuring the decision-making process through anonymous inputs, systematic scoring, and evidence-based methodologies. Butterfly reduces the weight of individual authority or reputation, ensuring that decisions are driven by the evaluations provided by participants, including their feedback and any supporting evidence submitted.

The purpose of this article is twofold: on the one hand, to present the results of the consensus reached on the use of corticosteroid therapy and, in particular, FP in nasal polyposis; on the other hand, to show how the use of artificial intelligence can be integrated in the decision-making process. This approach represents a methodological innovation and could have future applications in other clinical and scientific contexts.

## Materials and methods

**Figure 1:**
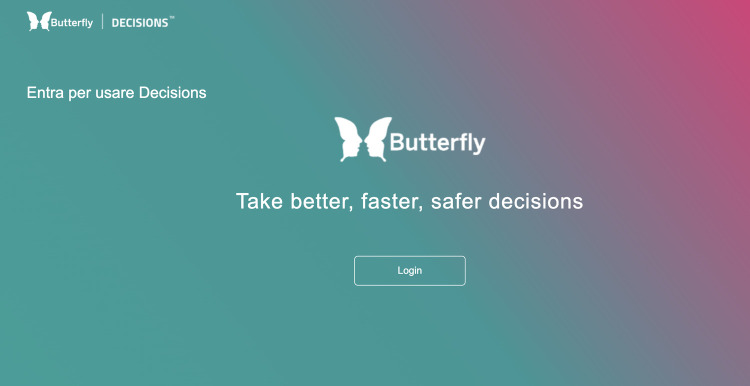
General screen of butterfly software.

The consensus analysis on using FP in the management of CRSwNP was conducted using the digital platform Butterfly Decisions (Butterfly DECISIONS s.r.l. via Francesco della Francesca 46, Salerno, Italy, https://butterflydecisions.com/?lang=en), designed to facilitate AI-driven decision-making processes (Figure 1). A multidisciplinary panel of experts evaluated 13 statements covering various aspects of managing CRSwNP, including diagnosis, use of topical and systemic corticosteroids, indications for biologic drugs, and surgical treatments. The experts involved in the consensus were affiliated with the leading Italian centers that are scientifically recognized for their expertise in rhinology.

The formulation of the statements was entrusted to the expert group during an in-person meeting, conducted according to a structured methodological approach and based on a careful review of the available scientific literature. The process began with a systematic review of the most relevant publications in the field of chronic rhinosinusitis with nasal polyposis (CRSwNP) and the use of topical corticosteroids, with a focus on international guidelines, including EPOS 2020, and already published randomised controlled trials. Sources were selected through targeted searches on PubMed and other scientific databases, favouring articles with a high level of evidence.

Once the data had been collected, the key information was synthesised and transformed into preliminary statements, which were formulated to accurately reflect the current state of the evidence and the main directions of the scientific community.

The platform used for the consensus, Butterfly Decisions, was based on a proprietary architecture called Monarch, which integrated generative artificial intelligence technologies with supervised and unsupervised machine learning models, with the aim of supporting decision-making among clinical experts in a structured and transparent manner.

**Figure 2:**
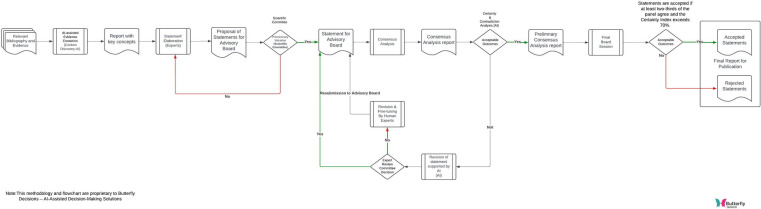
Workflow of the decision-making process used by the butterfly software.

The workflow employed in the study that utilizes context discovery and virtual assistants to create and, if necessary, revise statements within a consensus analysis process is showed in Figure 2.

**Figure 3:**
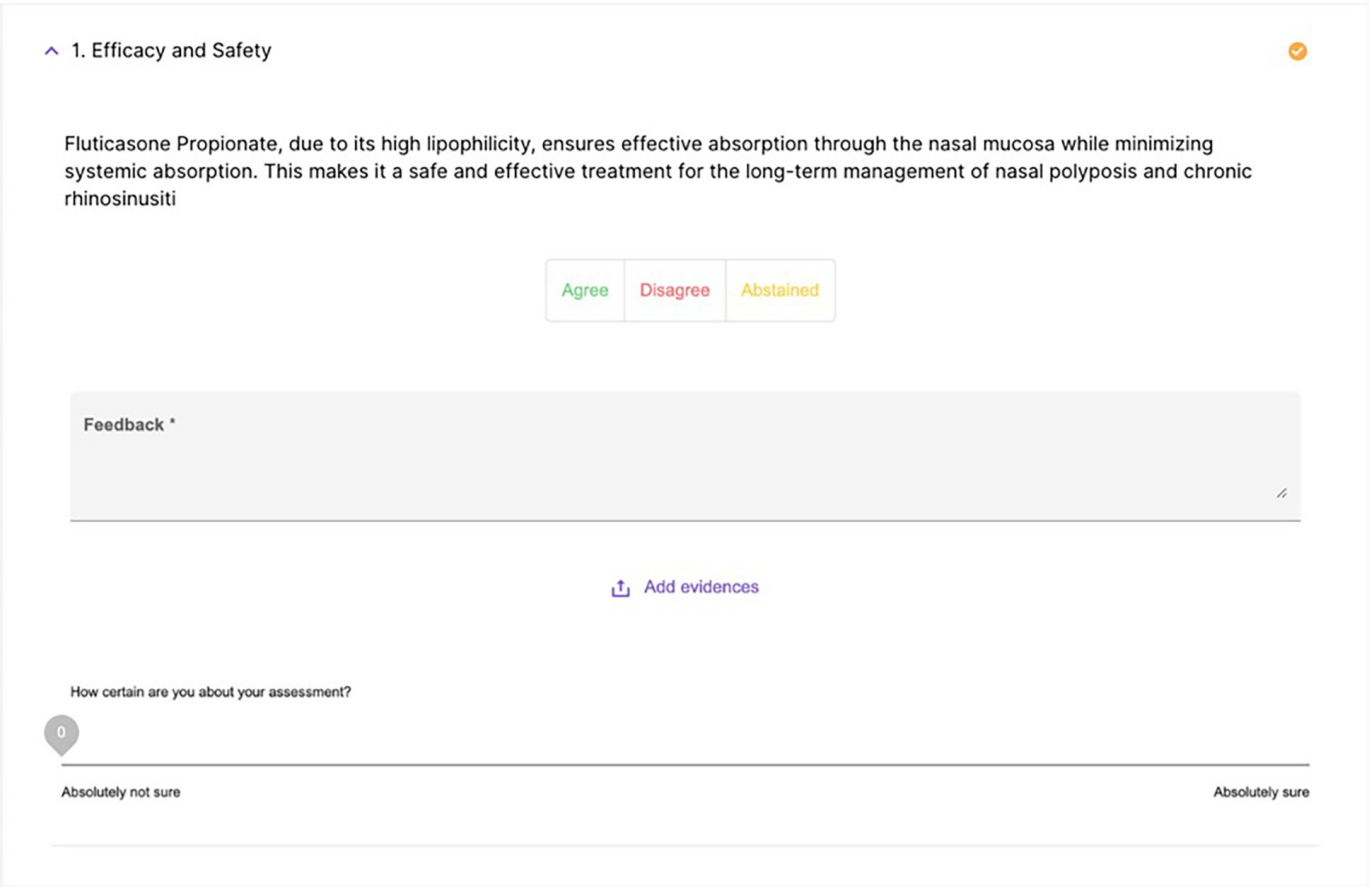
A screenshot of the text that the expert must evaluate, including options to agree, disagree, provide comments, and the certainty index.

The operation of the platform had several stages, starting with the automatic generation of preliminary statements, carried out by means of an AI-assisted semantic synthesis of scientific content uploaded on the platform or retrieved via PubMed. These statements were then subjected to evaluation by the experts online through a digital interface that allowed, for each statement, to express a binary vote (agree/disagree), indicate a confidence level on a continuous scale from 0 to 100, enter free textual comments and attach, where deemed useful, scientific references to support one's position (Figure 3).

Once the responses had been collected, Monarch ran an automated analysis based on two main indicators: Certainty Index (CertI) and the Contradiction Index (ContrI). The CertI measured the degree of support and consistency between favourable opinions, taking into account the proportion of experts in agreement, the mean and standard deviation of confidence levels, and the presence of abstentionist responses. The result was a summary value reflecting the extent to which a statement was supported by a strong and homogeneous consensus. Conversely, the ContrI quantified the degree of intra-group disagreement, combining the variability of binary responses, the dispersion in confidence levels and the possible presence of highly motivated minority subgroups. A high ContrI therefore indicated potentially controversial or polarised statements, even in the presence of an apparent majority.

In parallel, the platform had analysed textual comments using a semantic approach based on neural embedding models. The texts had been converted into semantic vectors, then projected into a reduced space using UMAP (Uniform Manifold Approximation and Projection), and finally clustered using HDBSCAN (Hierarchical Density-Based Spatial Clustering of Applications with Noise), with the aim of identifying coherent sets of similar remarks, areas of recurrent disagreement and shared suggestions. This had allowed the platform to automatically recognise emerging themes and propose changes to the statements, responding in a timely manner to the critical nodes identified.

In the event that a statement had shown a ContrI greater than 30%, the system had flagged it as controversial and triggered an automatic revision process. The proposed rewording could include terminological clarifications, the inclusion of significant bibliographic references (selected from those suggested by the experts) or structural changes aimed at reducing ambiguity and the possibility of misunderstanding. The new versions were then subjected to a further round of voting, maintaining full traceability of the version history, CertI and ContrI values associated with each iteration, and related comments.

To reduce the risk of systemic bias, the platform did not apply any filtering or automatic deletion of minority opinions. On the contrary, it ensured that discordant comments were retained, analysed and, where relevant, incorporated into subsequent submissions. Furthermore, all revisions suggested by the artificial intelligence were subjected to manual validation by the platform's editorial team before their final approval. This approach had made it possible to maintain transparency, traceability and methodological rigour at each stage of the consensus process, allowing a satisfactory level of agreement to be reached (CertI ≥ 80 and ContrI ≤ 30), without compromising the plurality of clinical opinions expressed.

The platform remained open for input over 16 days, providing clinicians ample opportunity to complete their evaluations. Any significant adverse events or observations from participant feedback were monitored and reported during the analysis, ensuring a safe and evidence-based evaluation. Finally, the results were integrated into a detailed report, validated by the scientific board, which provided a critical review of the 13 statements and updated recommendations for the management of CRSwNP.

## Results

**Figure 4:**
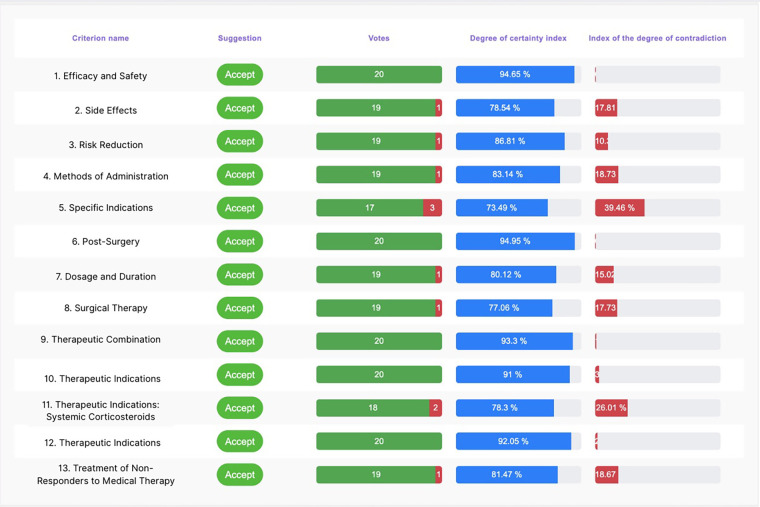
Dashboard of the statements.

The consensus conference was attended by 20 expert Italian otolaryngologists specializing in treating CRSwNP. During the event, 13 statements were presented, which, based on the degree of agreement and feedback received, were subsequently reviewed and approved by the board members. Remarkably, over 80% of participants completed their assessments within the first 72 hours. Six statements achieved a degree of agreement of 100%, six others 94.74% and one 84.21%. Below are the initial statements and the percentage of agreement, certainty, and contradiction, accompanied by the board members’ feedback and review of the statement. A dashboard summarized all the statements with the agreement, degree of certainty, and degree of contradiction (Figure 4).

### Statement 1: efficacy and safety

Proposed Statement: “Fluticasone Propionate, due to its high-fat solubility, ensures effective absorption through the nasal mucosa while minimizing systemic absorption. This makes it a safe and effective treatment for the long-term management of nasal polyposis and chronic rhinosinusitis.”

Agreement: 100%. Degree of certainty: 94.65%. Degree of contradiction: 0.71%.

Feedback: The participants noted no significant side effects, supported by scientific literature. However, one physician reported a potential reduction in efficacy in severe forms of nasal polyposis.

Consensus-Based Update: “Fluticasone Propionate, due to its high-fat solubility, ensures effective absorption through the nasal mucosa while minimizing systemic absorption. This makes it a safe and effective treatment for the long-term management of nasal polyposis and chronic rhinosinusitis. However, efficacy may vary in more severe forms of nasal polyposis” (7, 8).

### Statement 2: side effects

Proposed Statement: “Fluticasone Propionate has no side effects, given its low systemic bioavailability, in patients with glaucoma, hypertension and diabetes.”

Agreement: 95%. Degree of certainty: 78.54%. Degree of contradiction: 17.81%.

Feedback: Some participants emphasized the need to monitor intraocular pressure in patients with glaucoma, while others confirmed the drug's safety even in patients with pre-existing diseases.

Consensus-Based Update: “Fluticasone Propionate, due to its high-fat solubility, ensures effective absorption through the nasal mucosa while minimizing systemic absorption. Thanks to the low bioavailability related to the use of topical fluticasone, the risks of side effects in diabetic, hypertensive, and glaucoma patients are significantly low. This makes it a safe and effective treatment for the long-term management of nasal polyposis and chronic rhinosinusitis. However, efficacy may vary in more severe forms of nasal polyposis.” (7, 9, 10)

### Statement 3: risk reduction

Proposed Statement: “The fat-soluble formulation of Fluticasone Propionate is associated with a lower risk of systemic side effects than other intranasal corticosteroids, making it a preferred therapeutic choice for long-term treatment”.

Agreement: 95%. Degree of certainty: 86.81%. Degree of contradiction: 10.37%.

Feedback: Most participants agreed that FP is superior in efficacy and safety, while one physician expressed divergent opinions, citing the absence of significant differences compared to other corticosteroids, such as mometasone furoate.

Consensus-Based Update: “Fluticasone propionate is one of the topical molecules that provides high level of safety due to its minimal systemic bioavailability.” (PMID: 22448448).

Numerous articles in the literature, based on randomised controlled trials, report no serious adverse events in the use of Fluticasone Propionate (FP) (11–16).

### Statement 4: methods of application

Proposed Statement: “The efficacy of treatment with Fluticasone Propionate suspension varies significantly depending on the application method. Techniques such as Micro Aerosol Delivery (MAD), nasal douches, and high-volume nasal showers influence drug absorption and distribution, highlighting the importance of personalized treatment.”

Agreement: 95%. Degree of certainty: 83.14%. Degree of contradiction: 18.73%.

Feedback: The majority emphasized the importance of application techniques to maximize drug efficacy. However, one physician found no significant differences between the various administration methods.

Consensus-Based Update: “Fluticasone Propionate, due to its high-fat solubility, ensures effective absorption through the nasal mucosa while minimizing systemic absorption. The MAD device, nasal douches, and high-volume nasal showers impact drug absorption and distribution” (6, 17).

### Statement 5: specific indications analysis and review

Proposed Statement: “Discontinuation of Fluticasone Propionate is indicated in patients diagnosed with Chronic Rhinosinusitis.”

Agreement: 85%. Degree of Certainty: 73.49%. Degree of Contradiction: 39.46%.

Feedback: Most physicians agree that discontinuing FP is indicated for patients diagnosed with chronic rhinosinusitis. However, some physicians have suggested that temporary discontinuation and periodic checks are necessary, while others have shown that surgery may be the best option in severe chronic cases.

Consensus-Based Update: “Discontinuation of Fluticasone Propionate is indicated in patients diagnosed with chronic rhinosinusitis, with a personalized approach that may include temporary treatment discontinuation and periodic check-ups with an ENT specialist. A surgical option may be considered in cases of severe chronic rhinosinusitis”.

### Statement 6: post-surgery analysis and review

Proposed Statement: “Fluticasone Propionate post endoscopic sinus surgery (ESS) reduces the flare-up of chronic rhinosinusitis.”

Agreement: 100%. Degree of Certainty: 94.95%. Degree of Contradiction: 0.82%. Feedback: All physicians agreed that FP is effective in reducing flare-ups of chronic rhinosinusitis after ESS. However, some have stressed the importance of starting treatment only after surgical outcomes have stabilized.

Consensus-Based Update: “The use of Fluticasone Propionate post-operative ESS, once surgical outcomes have stabilized, significantly reduces the flare-up of chronic rhinosinusitis, improving symptom control and reducing the need for further intervention.” (18)

### Statement 7: dosage and duration analysis and review

Proposed Statement: “A dosage of 400 micrograms twice daily with a minimum duration of 12 weeks effectively reduces the volume of nasal polyps and improves the symptoms of chronic rhinosinusitis. Dosage and duration of treatment varies depending on the severity of the condition.”

Agreement: 95%. Degree of Certainty: 80.12%. Degree of Contradiction: 15.02%. Feedback: Most physicians agree that a dosage of 400*μ*g twice daily for a minimum of 12 weeks is effective. However, some physicians suggest customizing the dosage according to the severity of the disease and the patient's response.

Consensus-Based Update: “A dosage of 400 micrograms twice daily with a minimum duration of 12 weeks is generally effective in reducing the volume of nasal polyps and improving the symptoms of chronic rhinosinusitis. However, the dosage and duration of treatment can be customized according to the severity of the disease and individual patient response” (6, 19).

### Statement 8: surgical therapy analysis and review

Proposed Statement: “Medical therapy with topical corticosteroids, although effective in improving symptoms of nasal polyposis, is less effective than endoscopic surgery in controlling symptoms.”

Agreement: 95%. Degree of Certainty: 77.06%. Degree of Contradiction: 17.73%. Feedback: Most physicians agree that although topical corticosteroids effectively improve nasal polyposis symptoms, ESS offers superior symptom control. However, there are differences in opinion regarding the need to tailor treatment to the patient's specific case.

Consensus-Based Update: “Medical therapy with topical corticosteroids, although effective in improving the symptoms of nasal polyposis, is generally less effective than endoscopic surgery in controlling symptoms. However, both treatments have complementary roles in the overall management of CRSwNP, with topical corticosteroids often used as initial treatment and post-surgical maintenance therapy” (1).

### Statement 9: therapeutic combination analysis and review

Proposed Statement: “The combination of endoscopic surgery and topical therapy with Fluticasone Propionate significantly improves symptoms (nasal obstruction, reduced sense of smell, facial pain, etc.) in patients with chronic rhinosinusitis more effectively than medical therapy alone.”

Agreement: 100%. Degree of Certainty: 93.30%. Degree of Contradiction: 1.03%. Feedback: All physicians agree that the combination of ESS and topical therapy with FP offers significant improvement in the symptoms of chronic rhinosinusitis compared to medical therapy alone. Comments suggest that combining these treatments leads to a more effective and lasting management of symptoms.

Consensus-Based Update: “The combination of endoscopic surgery and topical therapy with Fluticasone Propionate significantly improves symptoms in patients with chronic rhinosinusitis more effectively than medical therapy alone. Surgery facilitates access of topical drugs to diseased areas, and topical corticosteroids help maintain the benefits of surgery, reducing the risk of recurrence and improving patients’ quality of life” (20, 21).

### Statement 10: therapeutic indications

Proposed Statement: “Topical corticosteroids, such as Fluticasone Propionate, are the therapy of first choice in patients with no indication for treatment with biological drugs, according to guidelines.”

Agreement: 100%. Degree of Certainty: 91.00%. Degree of Contradiction: 3.24%. Feedback: All physicians agree that topical corticosteroids, such as FP, are the therapy of first choice for patients with chronic rhinosinusitis without indications for treatment with biologic drugs, as indicated by the guidelines.

Consensus-Based Update: “Topical corticosteroids, such as Fluticasone Propionate, are the therapy of first choice in patients with chronic rhinosinusitis with nasal polyposis, in the absence of indications for treatment with biological drugs and patients without comorbidities such as asthma or NSAIDs and therefore according to the guidelines. These drugs effectively reduce symptoms and improve patients’ quality of life, with a favorable safety profile” (1).

### Statement 11: therapeutic indications: systemic corticosteroids

Proposed Statement: “Therapy with topical corticosteroids, such as Fluticasone Propionate, in combination with systemic corticosteroids is most effective in patients in whom there is no indication for treatment with biological drugs, according to guidelines.”

Agreement: 90%. Degree of Certainty: 78.30%. Degree of Contradiction: 26.01%.

Feedback: Most physicians agreed that combining topical and systemic corticosteroids is effective in patients for whom treatment with biologic drugs is not indicated. However, some expressed concerns about side effects and prolonged use of systemic corticosteroids.

Consensus-Based Update: “Therapy with topical corticosteroids, such as Fluticasone Propionate, in combination with systemic corticosteroids may be more effective in patients with no indication for treatment with biologic drugs, according to the guidelines. However, systemic corticosteroids should be limited to short periods to reduce the risk of side effects” (1).

### Statement 12: therapeutic indications—biological drugs

Proposed Statement: “Biologic drugs are indicated in patients who are non-responders to topical and systemic corticosteroid therapy and who meet the requirements of current guidelines.”

Agreement: 100%. Degree of Certainty: 92.05%. Degree of Contradiction: 2.11%. Feedback: All physicians agreed on the indication of biological drugs for non-responders to topical and systemic corticosteroid therapy. Some comments highlighted specific situations and clarifications related to the use of biological drugs, with a firm reference to the guidelines.

Consensus-Based Update: “Biologic drugs are indicated in patients who are non-responders to topical and systemic corticosteroid therapy and meet current guidelines’ requirements. It is also important to consider patients with relapses after surgical therapy and those not amenable to surgical therapy” (1).

### Statement 13: treatment of non-responders to medical therapy

Proposed Statement: “Surgical treatment should be considered in patients with chronic rhinosinusitis who do not respond adequately to medical therapy with topical and systemic corticosteroids and in patients already treated with surgery and non-responders to biological drugs.”

Agreement: 90%. Degree of Certainty: 81.47%. Degree of Contradiction: 18.67%. Feedback: All physicians agreed to consider surgery for non-responders to medical therapy, including those non-responders to biological drugs after surgery. All physicians agreed on the importance of considering surgery for patients who do not respond to medical treatment with topical and systemic corticosteroids and for those who do not respond to biologic drugs despite prior surgery (22).

Table 1 summarises the statistical values and the index of certainty and uncertainty for each statement.

**Table 1:** The table shows for each statements, the total number of respondents (n.), how many agreed or disagreed, the standard descriptive statistics: mean agreement in percentage, mean confidence, standard deviation (Std. Dev.), inter quartile range (IQR) and the Certainty and Contradiction indices.

Statement	n.	Agrees	Disagrees	Mean agreement	Mean confidence	Std. Dev. (Conf.)	IQR (Conf.)	Certainity index	Contrddiction index
1	20	20	0	100.0%	99.60	24.95	8.50	94.65%	0.71%
2	20	19	1	95.0%	88.35	13.98	12.00	78.54%	17.81%
3	20	19	1	95.0%	89.25	15.80	15.50	86.81%	10.37%
4	20	19	1	95.0%	93.45	10.50	14.50	83.14%	18.73%
5	20	17	3	85.0%	85.60	18.20	25.00	73.49%	39.46%
6	20	20	0	100.0%	90.85	11.50	13.00	94.95%	0.82%
7	20	19	1	95.0%	86.90	14.30	17.00	80.12%	15.02%
8	20	19	1	95.0%	84.70	17.90	22.50	77.06%	17.73%
9	20	20	0	100.0%	91.40	12.70	15.00	93.3%	1.03%
10	20	20	0	100.0%	86.25	16.10	18.00	91%	3.24%
11	20	18	2	90.0%	85.55	14.80	15.50	78.3%	26.01%
12	20	20	0	100.0%	89.60	9.40	10.00	92.05%	2.11%
13	20	18	2	90.0%	88.15	13.20	16.00	81.47%	18.67%

## Discussion

The decision to focus the consensus on the use of FP in patients with CRSwNP was dictated by several clinical and pharmacological considerations. Due to its high-fat solubility, FP has a reduced risk of systemic absorption, making it particularly suitable for long-term treatment (7, 8). It exerts an effective local action on the nasal mucosa, associated with reduced systemic bioavailability, limiting the risk of systemic side effects. All board members agreed on this point, stressing that the benefits are reduced due to massive polyposis. Although FP has a low systemic bioavailability, it is not entirely without potential side effects. Nasal corticosteroids, such as Fluticasone, may affect intraocular pressure, although reported cases of glaucoma are rare and generally associated with high dosages or prolonged use. Patients with glaucoma must regularly monitor their eye pressure during treatment with this drug (9).

An expert's opinion on the systemic side effects of fluticasone was associated with a 17.81% level of contradiction in statement 2. In contrast, FP is applied topically and has minimal bioavailability. Butterfly's AI-based system identified this misunderstanding, reducing the impact of the contradiction.

About hypertension, nasal corticosteroids such as Fluticasone do not significantly increase blood pressure. It can be considered safe for hypertensive patients due to its low systemic bioavailability, which limits interaction with the cardiovascular system.

In the case of diabetes, there is no direct evidence to suggest a significant risk of worsening glycaemic control using nasal fluticasone (10). However, careful surveillance is always advisable, especially in diabetic patients, who may be more susceptible to the systemic effects, even if minimal, of corticosteroids. Nevertheless, at doses of 400μg twice a day, no alterations in the hypothalamic-pituitary-adrenal axis were observed (7). These characteristics make it an optimal therapeutic choice for treating chronic rhinosinusitis and nasal polyposis, providing effective symptom control and a lower risk of systemic complications than other corticosteroids with higher systemic bioavailability. The discussion around the “discontinuation of FP” arose due to divergent clinical opinions regarding the duration of treatment. Some experts suggested that discontinuing or tapering therapy might be appropriate for patients in stable remission to reduce side effects or address patient preferences. This led to higher contradiction and lower certainty for this statement, as some preferred indefinite maintenance, while others supported periodic reassessment. Despite the moderate disagreement, the majority of experts accepted the statement, which met the predefined acceptance criteria.

Advanced delivery systems, such as MAD and High Volume Steroid Nasal Rinse (HSNR) nasal irrigations, significantly improve drug delivery and penetration into the nasal cavities (6, 17). These approaches are efficient in the postoperative period, e.g., after ESS, by facilitating a more targeted and uniform action on the mucous membranes and helping to optimize therapeutic outcomes. Applying topical corticosteroids in the postoperative period significantly improves symptoms, reduces the recurrence and volume of nasal polyps, and significantly improves patients’ quality of life (18, 19, 23). The commonly accepted dosage for FP in postoperative treatment, mainly after ESS, is 400μg twice daily for 10–12 weeks, which can be adjusted according to the patient's clinical condition and response to treatment (6, 19).

The use of topical FP in patients with CRSwNP is effective in reducing the need for surgery compared to placebo (24). However, in patients who do not respond adequately to medical therapy, resolution of the pathology requires a surgical approach (1). In addition, topical corticosteroids, such as FP, are considered the therapy of first choice in patients with CRSwNP who do not indicate treatment with biologic drugs and patients without comorbidities such as asthma or NSAIDs even with the addition of systemic corticosteroid therapy, as recommended by guidelines (1).

FP is indicated as a first-line therapy for a large group of patients without the comorbidities mentioned above that make CRSwNP “complicated.” This population represents approximately 43% of individuals affected by CRSwNP (25). Therefore, a high-efficacy-low-bioavailability topical corticosteroid like FP is considered the primary therapeutic choice.

The moderate contradiction observed in Statement 11 regarding the use of systemic steroids in CRSwNP reflects the variability in clinical practice regarding their use. The main concern stems from the potential side effects associated with systemic steroids, such as hyperglycaemia, hypertension and adrenal suppression. Although some clinical guidelines, such as those of EPOS (1), recommend short courses or “rescue therapies” with systemic steroids for acute exacerbations, there may still be concern about the risks. This divergence in practice probably contributed to the 26% contradiction index. Nevertheless, most experts agree that short-term systemic steroid use remains useful for managing severe exacerbations.

Biological drug therapy is reserved for patients who cannot be stabilized with topical treatment or who require repeated courses of systemic therapy, even though they have already undergone surgery. In addition, it is indicated for patients unsuitable for surgery (1). Surgical treatment should be considered in patients with chronic rhinosinusitis who do not respond adequately to medical therapy. In addition, surgery may also be indicated in patients who have been treated surgically in the past but are not suitable to biological therapy. In these cases, surgery may help remove nasal polyps and improve sinus drainage, offering an option for symptom control and improving quality of life (1). It is useful to note that statements 5, 11 and 13 recorded a consensus of 85%–90%, with one to three dissenters, but the algorithm did not require a second review. According to the current Butterfly Decisions protocol, statements with a consensus of more than 85%–90% and a Certainty Index ≥70% are considered sufficiently supported, even in the presence of dissent. Although the Contradiction Index indicated heterogeneity, the majority consensus was strong enough not to trigger an automatic review. In future studies, we may review statements with a high Contradiction Index to further explore minority positions. In addition, we are considering an approach that would provide for additional review if there was both a strong consensus and a high Contradiction Index, to address any unresolved bias. Optional textual comments provided by participants enriched the review and helped to acknowledge and address any disagreements in subsequent rounds. The AI platform Butterfly Decisions, structuring the decision-making process around granular participant input, allowed for collecting and integrating detailed evaluations, feedback, and supporting evidence from the expert panel. This process mitigated potential biases and ensured each contribution was reasonably and systematically considered. Butterfly Decisions also streamlined the entire process through its dynamic dashboards, which updated in real-time as feedback was received. Over 80% of physicians completed their assessments within the first 3 days. This efficiency extended to the medical writing phase, as the platform leveraged pre-trained AI models specifically designed for clinical writing and analysis. These tools facilitated the rapid generation of high-quality drafts, enabling faster review and revision of the consensus statements. The integration of AI tools and real-time feedback mechanisms not only improved the transparency and objectivity of the consensus process but also demonstrated a significant potential to save time and resources.

## Conclusions

The consensus stated that FP is a first-line treatment option, as the other topical corticosteroids, for patients with CRSwNP who do not have comorbidities that could complicate their clinical condition. Its high efficacy and low systemic bioavailability make it suitable for long-term use (7, 8).

Topical FP has been shown to limit disease progression and decrease the need for surgical intervention (25). Advanced drug delivery systems enhance drug distribution on the nasal mucosa (6, 17). This approach reduces the risk of polyp recurrence and improves patients’ quality of life (18, 19, 23).

If topical therapy, even when combined with short courses of systemic steroids, is insufficient, biological therapies or surgical options should be considered (1). ESS is a valid option for patients who do not adequately respond to conventional medical therapy or who experience recurrences that cannot be controlled even with biological treatments. Due to its proven safety and efficacy, FP remains a cornerstone in managing uncomplicated CRSwNP, allowing for the postponement or, in some cases, the avoidance of more invasive therapeutic solutions. A limitation of this study was the absence of stratification of patients with CRSwNP according to specific phenotypes and enotypes, such as eosinophilic or non-eosinophilic and aspirin-exacerbated respiratory disease (AERD), future studies are needed to assess the influence of these factors to improve treatment customisation.

A key strength of this study was the use of Butterfly Decisions. As a recommendation, future efforts should continue refining AI models within the platform, emphasizing further specialization in clinical decision support and medical research. By doing so, Butterfly Decisions can continue to enhance its contribution to advancing evidence-based clinical practices.

## Data Availability

The original contributions presented in the study are included in the article/Supplementary Material, further inquiries can be directed to the corresponding author.
